# Differential Methylation as a Biomarker of Response to Etanercept in Patients With Rheumatoid Arthritis

**DOI:** 10.1002/art.39590

**Published:** 2016-04-21

**Authors:** Darren Plant, Amy Webster, Nisha Nair, James Oliver, Samantha L. Smith, Steven Eyre, Kimme L. Hyrich, Anthony G. Wilson, Ann W. Morgan, John D. Isaacs, Jane Worthington, Anne Barton

**Affiliations:** ^1^ NIHR Manchester Musculoskeletal Biomedical Research Unit, Manchester Academy of Health Sciences, and Central Manchester NHS TrustManchesterUK; ^2^ Arthritis Research UK Centre for Genetics and Genomics, University of ManchesterManchesterUK; ^3^ University College Dublin School of Medicine and Medical Science, and Conway InstituteDublinIreland; ^4^ NIHR Leeds Musculoskeletal Biomedical Research Unit, Leeds Teaching Hospitals NHS Trust and Leeds Institute of Rheumatic and Musculoskeletal Medicine, University of LeedsLeedsUK; ^5^ NIHR Newcastle Biomedical Research Centre in Ageing and Chronic Disease, Newcastle University, and Newcastle‐Upon‐Tyne NHS Foundation TrustNewcastle‐Upon‐TyneUK; ^6^ NIHR Manchester Musculoskeletal Biomedical Research Unit, Manchester Academy of Health Sciences, and Central Manchester NHS Trust, and Arthritis Research UK Centre for Genetics and Genomics, University of ManchesterManchesterUK

## Abstract

**Objective:**

Biologic drug therapies represent a huge advance in the treatment of rheumatoid arthritis (RA). However, very good disease control is achieved in only 30% of patients, making identification of biomarkers of response a research priority. We undertook this study to test our hypothesis that differential DNA methylation patterns may provide biomarkers predictive of response to tumor necrosis factor inhibitor (TNFi) therapy in patients with RA.

**Methods:**

An epigenome‐wide association study was performed on pretreatment whole blood DNA from patients with RA. Patients who displayed good response (n = 36) or no response (n = 36) to etanercept therapy at 3 months were selected. Differentially methylated positions were identified using linear regression. Variance of methylation at differentially methylated positions was assessed for correlation with *cis‐*acting single‐nucleotide polymorphisms (SNPs). A replication experiment for prioritized SNPs was performed in an independent cohort of 1,204 RA patients.

**Results:**

Five positions that were differentially methylated between responder groups were identified, with a false discovery rate of <5%. The top 2 differentially methylated positions mapped to exon 7 of the *LRPAP1* gene on chromosome 4 (cg04857395, *P* = 1.39 × 10^−8^ and cg26401028, *P* = 1.69 × 10^−8^). The A allele of the SNP rs3468 was correlated with higher levels of methylation for both of the top 2 differentially methylated positions (*P* = 2.63 × 10^−7^ and *P* = 1.05 × 10^−6^, respectively). Furthermore, the A allele of rs3468 was correlated with European League Against Rheumatism nonresponse in the discovery cohort (*P* = 0.03; n = 56) and in the independent replication cohort (*P* = 0.003; n = 1,204).

**Conclusion:**

We identify DNA methylation as a potential biomarker of response to TNFi therapy, and we report the association between response and the *LRPAP1* gene, which encodes a chaperone of low‐density lipoprotein receptor–related protein 1. Additional replication experiments in independent sample collections are now needed.

Rheumatoid arthritis (RA) is a common autoimmune disorder affecting up to 1% of individuals in Western populations. The treatment of RA traditionally consisted of corticosteroids and antiinflammatory drugs to treat symptoms in combination with disease‐modifying antirheumatic drugs (DMARDs) to slow disease progression 1. In 1998 the introduction of biologic drug therapies provided a new form of treatment. While these therapies have proved effective for many patients, very good disease control or remission is achieved in only up to 30% of patients 2. In the time taken to find which therapy is effective for a given patient, progression of disease and accumulation of disability can have a negative impact on the quality of life. This makes the identification of biomarkers predictive of response a research priority. Such biomarkers would allow implementation of stratified medicine to better target available therapies to those patients most likely to respond to them.

Response to biologic agents is complex and is influenced by sex, concurrent use of DMARDs, and level of disease activity and severity 3, 4, 5, 6, 7. Two recent investigations have identified genetic markers that are robustly associated with response to tumor necrosis factor inhibitor (TNFi) therapy. First, a genome‐wide association study (GWAS) meta‐analysis including 2,706 RA patients identified a single‐nucleotide polymorphism (SNP) (rs6427528) in the *CD84* gene as a predictor of response to etanercept in European patients (*P* = 8 × 10^−8^; n = 733) 8. This was shortly followed by the identification of a SNP (rs3794271) mapping to the *PDE3A‐SLC01C1* locus that reached genome‐wide significance following meta‐analysis of data from Spanish and Danish RA cohorts (*P* = 3.3 × 10^−10^) 9. However, even when all known predictive factors are combined, the proportion of variance in treatment response accounted for remains modest 10.

Epigenetics is a broad term that describes heritable features which alter gene expression without altering the underlying DNA sequence 11. DNA methylation is one form of epigenetic modification and is an attractive candidate for investigation as a biomarker of treatment response as it is relatively stable compared to messenger RNA and most proteins, is altered by drug therapy, and is amenable to high‐throughput whole‐genome typing.

We hypothesized that differential DNA methylation might provide a useful biomarker predictive of response to TNFi therapy in RA. Therefore, we performed an epigenome‐wide association study to identify differential methylation between good responders and nonresponders among patients with RA being treated with etanercept. We elected to test whole‐genome blood samples, as a biomarker identified in that type of sample would be most readily translated to clinical benefit.

## PATIENTS AND METHODS

### Patient selection and sample preparation

Patients were recruited to the Biologics in Rheumatoid Arthritis Genetics and Genomics Study Syndicate (BRAGGSS) from 57 UK centers 12. All patients were Caucasian, age ≥18 years, and clinically classified at inclusion as having RA according to the 1987 revised criteria of the American College of Rheumatology 13. Clinical and biologic samples were collected prior to treatment with biologic drugs, and patients were then followed up prospectively with data and sample collection at 3, 6, and 12 months after initiation of therapy. We selected patients for the current study who had been treated with etanercept and who had had an extreme response phenotype at 3 months—either a good response, defined as a Disease Activity Score in 28 joints (DAS28) 14 of <2.6, or nonresponse, defined as an improvement in the DAS28 of <0.6 or as an end point DAS28 of >5.1 15. DNA was extracted from pretreatment whole blood samples and stored at −80°C.

### Epigenome‐wide association study

For each pretreatment DNA sample, 500 ng DNA was bisulfite converted using EZ DNA methylation kits according to the amended protocol of the manufacturer (Zymo Research) for use with an Infinium Methylation Assay (Illumina). Epigenome‐wide methylation was assessed with the Infinium HD Methylation Assay according to the protocol recommended by the manufacturer, using HumanMethylation450 BeadChips. The DNA was denatured and neutralized, then isothermally amplified overnight. The amplified product was enzymatically fragmented, then precipitated using isopropanol. After resuspension, the fragmented DNA sample was transferred to the BeadChips and incubated overnight in a hybridization oven. Twelve samples were hybridized to each BeadChip, which is formatted as a 6 × 2 matrix. DNA from responders and nonresponders was aliquoted onto alternate rows and columns of the BeadChips to reduce any bias that might result from the position on the BeadChip. After washing nonhybridized DNA from the BeadChip, capillary flow‐through chambers were used to perform single base extension and staining of the DNA. The BeadChips were then imaged using an Illumina iScan System.

### Data analysis of methylation arrays

All data analysis was performed in R 3.2.0 (www.r-project.org) 16. Data quality for each sample was assessed by visual inspection of control probe summary data in GenomeStudio (Illumina), by visual inspection of kernel density plots of methylation beta values, and by comparing median log_2_ intensities recorded in both the methylated and unmethylated channels. If both the methylated and unmethylated channels recorded background signal levels at the detection threshold of *P* > 0.01, then the data for that probe were excluded from further analysis 17. In addition, probes containing SNPs in the CpG interrogation site or single‐nucleotide extension site were excluded along with cross‐reactive probes (i.e., those that cohybridize to alternate genomic sequences). Raw beta values were logit transformed to M values 18 following subset‐quantile within array normalization (SWAN) 19, and principal components analysis (PCA) was used to assess potential batch effects, including position on BeadChip. Batch effect correction was performed using published empirical Bayes methods 20, and distribution of cell types within the whole blood sample was inferred for each patient using published methods 21. Linear regression models were used to test for association between CpG methylation and treatment response 22. Baseline disease activity, sex, age, concurrent use of DMARDs, and cell type composition were included in the model as covariates. Power to detect differential DNA methylation between responder groups was estimated using calculations presented in ref. 23.

### Technical validation of array data using pyrosequencing

A custom pyrosequencing assay was designed to target the top differentially methylated position from the BeadChip data. The assay was designed using PyroMark Assay Design software 2.0 (Qiagen). Ten nanograms of template bisulfite‐converted DNA was added to 1× PyroMark PCR Master Mix (Qiagen), 1× CoralLoad Concentrate (Qiagen), and 0.2 μ*M* PCR primer set (Qiagen). The solution was made up to 25 μl with RNase‐free water (Qiagen) and was amplified in a thermocycler (DNA Engine Dyad; MJ Research) using polymerase chain reaction (PCR) conditions recommended by the manufacturer (Qiagen).

The remaining PCR product (10–20 μl) was agitated at 1,400 revolutions per minute for 10 minutes with 2 μl streptavidin–Sepharose High Performance Beads (GE Healthcare) and 40 μl PyroMark Binding Buffer (Qiagen) made up to 80 μl with Milli‐Q water. The samples were applied to the vacuum handset of the PyroMark Q24 workstation and washed with 70% ethanol, PyroMark denaturation solution, and PyroMark wash buffer (Qiagen) before being added to 0.3 μ*M* sequencing primer diluted to 25 μl in annealing buffer (Qiagen). To allow the biotin‐labeled DNA strand to anneal to the primers, the solution was heated to 80°C for 2 minutes, then allowed to cool to room temperature for 5 minutes. Pyrosequencing reactions were performed using a PyroMark Q24 and PyroMark Gold Q24 Reagents (Qiagen).

### Correlating DNA methylation level with genetic markers

Fifty‐six of the 72 patient samples analyzed for DNA methylation had previously been genotyped on the OmniExpress genotyping array (Illumina). The array targets >700,000 SNPs with genome‐wide coverage. Array data were processed for quality in Plink 24 using previously described methods 25. Briefly, closely related individuals, individuals with a poor call rate (>2% missing data), and ethnic outliers were removed, as were SNPs with >2% missing data, minor allele frequency (MAF) <1%, or departing from Hardy‐Weinberg equilibrium with a *P* value less than 5 × 10^−7^. SNPs passing quality assessment were imputed using the 1,000 Genomes reference panel (https://mathgen.stats.ox.ac.uk/impute/data_download_1000G_phase1_integrated.html) and IMPUTE2 software 26. Following imputation, SNPs with an imputation “info” score <0.9, >2% missing data, MAF <1%, or a Hardy‐Weinberg equilibrium *P* value less than 5 × 10^−7^ were removed. Only those SNPs mapping to within 1 million bases of the most compelling differentially methylated CpG sites were tested for *cis*‐acting methylation quantitative trait locus (QTL) analysis 27.

In addition, independent of the 56 patients described above, 1,204 patients treated with TNFi (36 with certolizumab, 372 with adalimumab, 414 with etanercept, and 382 with infliximab) had both genome‐wide SNP and European League Against Rheumatism (EULAR) response 28 data available for replication analysis. The vast majority of these DNA samples were collected after treatment, making them suitable for SNP analysis but not for methylation analysis. Genetic data for the replication experiments were generated across multiple arrays (Affymetrix 5.0 [n = 552], Affymetrix 6.0 [n = 243], OmniExpress [n = 409]), and each array was processed for quality control using the methods described above. SNP data for variants showing evidence of association in the discovery cohort (n = 56) were extracted and tested for association in the replication samples to avoid the issue of multiple testing.

## RESULTS

### Cohort characteristics

Patient samples were selected from the BRAGGSS, a prospective longitudinal study of response to biologic therapies in patients with RA. At the beginning of the current study, 890 patients had a pretreatment DNA sample available, and 583 of those patients had reached 3 months of follow‐up and were eligible for treatment response studies. Two hundred nine of those patients were treated with etanercept, and 89 of the 209 patients either responded well (n = 39) or failed to respond (n = 50) to therapy at 3 months, based on established EULAR response criteria. From those 89 patients, pretreatment DNA samples were selected for 36 very good responders (i.e., with clinical remission of their disease) and 36 nonresponders. Baseline characteristics of the 2 groups are summarized in Table 1. No statistically significant differences were observed between treatment response groups when comparing age, DAS28, concurrent DMARD therapy, disease severity, or disease duration. However, good responders tended to be younger and to have less disability and longer disease duration. This study had 80% power to detect a mean methylation difference of 7% between good responders and nonresponders at the 5% significance threshold.

**Table 1:** Baseline characteristics of the patients selected for the epigenome‐wide association study of response to etanercept in patients with rheumatoid arthritis^a^

Characteristic	Good responders (n = 36)	Nonresponders (n = 36)
Age, mean ± SD years	54.6 ± 11.4	59.9 ± 12.2
Women, no. (%)	28 (78)	29 (81)
DAS28 at baseline, mean ± SD	5.9 ± 1.2	5.8 ± 0.76
Concurrent DMARD therapy, no. (%)	31 (86)	32 (88)
Receiving methotrexate as DMARD, no. (%)^b^	21 (78)	25 (78)
HAQ score at baseline, median (IQR)	1.5 (1.25–2)	2.0 (1.6–2.6)
Disease duration at baseline, median (IQR) months	11.3 (5.1–21.8)	7.2 (2.8–11.5)

a No statistically significant differences were observed between the treatment response groups. DAS28 = Disease Activity Score in 28 joints; HAQ = Health Assessment Questionnaire; IQR = interquartile range.

b Data on specific disease‐modifying antirheumatic drugs (DMARDs) were available for 27 responders and 32 nonresponders.

### Epigenome‐wide association study findings

**Figure 1:**
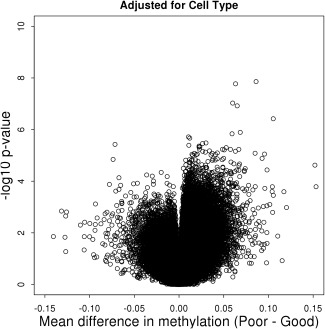
Volcano plot of −log_10_ *P* value versus mean difference in methylation. CpG positions that are more methylated in nonresponders compared to good responders are plotted to the right.

Following visual assessment of kernel density plots of methylation beta values and plots of log median (M) and (U) intensities, 1 sample with an aberrant methylation profile was identified and removed from subsequent analyses. In the remaining samples, cross‐reactive probes along with probes that failed detection or contained SNPs in the probe sequence were removed. A total of 422,638 probes were available for further analyses in 71 patients (35 nonresponders and 36 good responders). Methylation data were further processed using SWAN and logit transformed prior to PCA. PCA revealed a relationship between position on the BeadChip and the first principal component (*P* = 9.5 × 10^−9^). Since position on the BeadChip was anticipated as a potential batch effect, biologic samples were distributed on the chip in such a way as to reduce any bias this might cause (see Patients and Methods), and the methylation data were adjusted for position effects using an empirical Bayes method. The largest mean difference in methylation observed between responder groups was ∼15%, with more methylation observed on average in nonresponders compared to good responders for probes with smaller *P* values (Figure 1).

**Figure 2:**
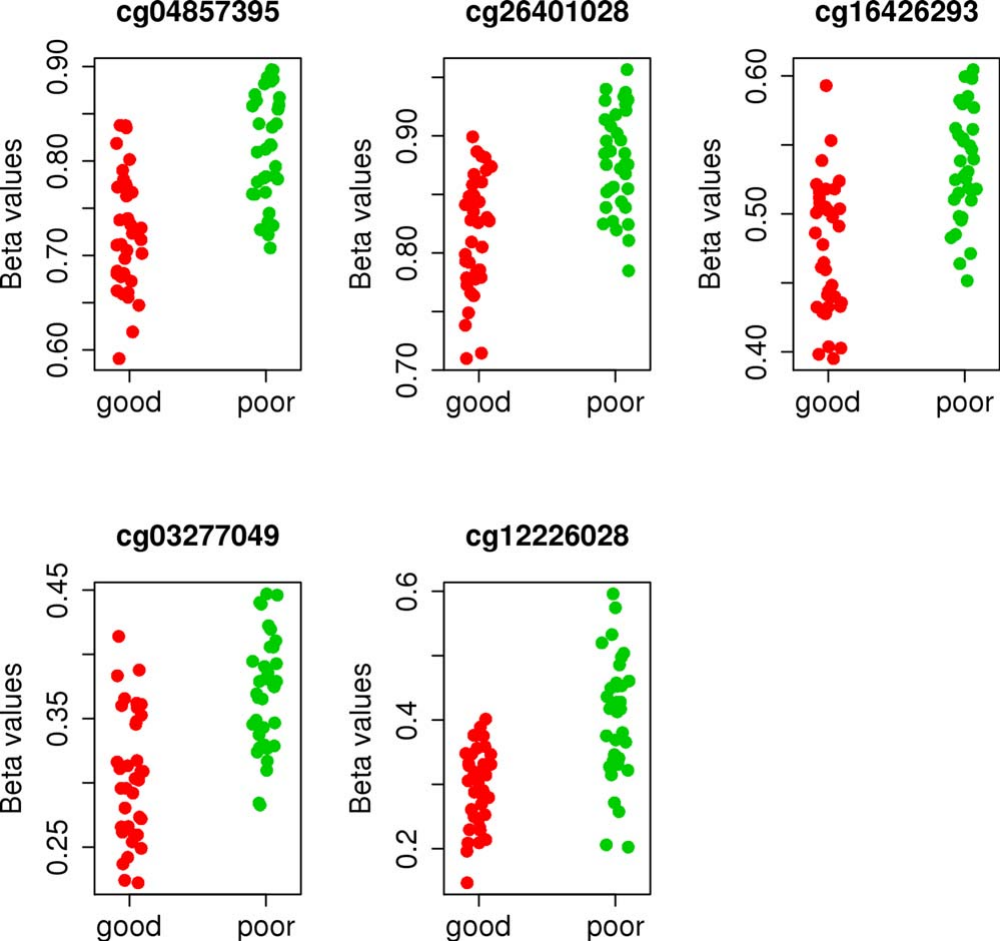
Plot of CpG methylation values for the top 5 differentially methylated positions in both good responders and poor responders (i.e., nonresponders). Color figure can be viewed in the online issue, which is available at http://onlinelibrary.wiley.com/journal/doi/10.1002/art.39590/abstract.

Five differentially methylated positions were correlated with treatment response, with a false discovery rate (FDR) of <5%. The 5 differentially methylated positions showed more methylation in nonresponders than in good responders (Table 2 and Figure 2). No difference in composition of cell type was observed between responder groups. Furthermore, adjustment for differential cell counts did not have a strong impact on the results when compared to the unadjusted analysis (additional information is available upon request from the corresponding author).

**Table 2:** Positions differentially methylated between responders and nonresponders to etanercept, identified using HumanMethylation450 BeadChips

Probe ID	Good responders, mean % methylation	Nonresponders, mean % methylation	*P*	*P*, adjusted
cg04857395	72	81	1.39 × 10^−8^	0.004
cg26401028	82	88	1.69 × 10^−8^	0.004
cg16426293	48	54	9.41 × 10^−8^	0.01
cg03277049	30	37	1.21 × 10^−7^	0.01
cg12226028	30	40	3.81 × 10^−7^	0.03

**Figure 3:**
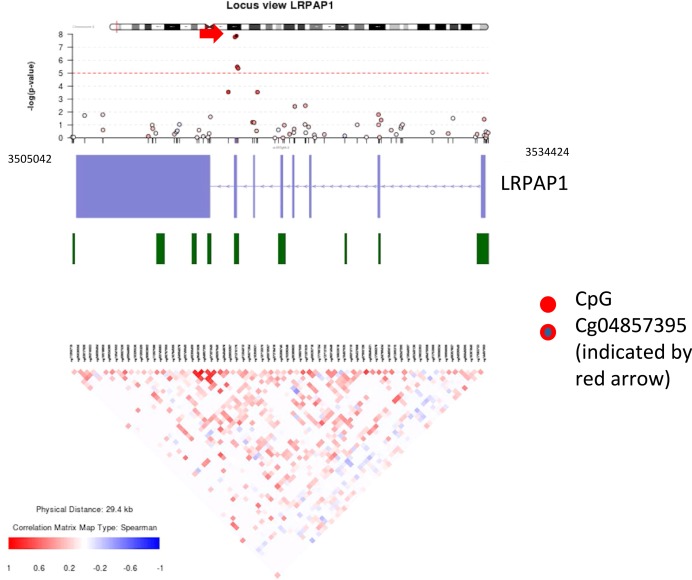
Locus view of the *LRPAP1* region on chromosome 4. Top, Plot of −log_10_ *P* value (y‐axis) versus position (x‐axis). The red line indicates the value of 5 × 10^−8^ (genome‐wide significance threshold). Middle, Schematic drawing of gene and position of CpG islands. Bottom, Correlation structure of DNA methylation at the CpG sites in the genomic region.

Two of the top 5 CpG and 4 of the top 15 CpG (ranked 1st, 2nd, 10th, and 14th) were located within exon 7 of the *LRPAP1* gene on chromosome 4 (Figure 3) (further information available upon request from the corresponding author). All 4 CpG positions were more methylated in the nonresponder group than in the good responder group.

### Validation and replication of epigenome‐wide association study findings using pyrosequencing

For 38 patient samples, a sufficient quantity of bisulfite‐converted DNA to permit technical validation of the BeadChip data remained available following the BeadChip experiment. For the most differentially methylated position, cg04857395, the Spearman's rank correlation coefficient was 0.87, demonstrating a high degree of correlation between the 2 platforms (additional information available upon request from the corresponding author).

### Methylation QTL/genetic analysis

The *LRPAP1* locus was prioritized for methylation QTL analysis as this locus revealed the most compelling evidence for differential methylation between the TNFi responder groups. Three SNPs, rs3468, rs6850908, and rs1049574, were correlated with methylation levels at positions cg04857395 and cg26401028 with an FDR of <5%. SNPs rs6850908 and rs1049574 were highly correlated with one another (R^2^ = 0.85) but were less strongly correlated with rs3468 (R^2^ <0.45). All 3 SNPs were genotyped on the array (i.e., not imputed). The A allele of rs3468 was correlated with higher methylation levels for both probe positions with an FDR‐adjusted *P* value of <0.007 (Table 3) (additional information available upon request from the corresponding author). The A allele of rs3468 was also correlated with EULAR nonresponse in the 56 samples (32 good responders, 24 nonresponders) analyzed for DNA methylation, with an odds ratio of 2.9 (95% confidence interval [95% CI] 1.11–7.37) (*P* = 0.03). SNP rs3468 was further analyzed in an independent sample of 1,204 TNFi‐treated patients (additional information available upon request from the corresponding author). Here, the ordered logistic regression model revealed that a 1‐unit increase in the rs3468 genotype (i.e., GG to GA or GA to AA) resulted in a 1.28‐fold increase (95% CI 1.08–1.49) in the odds of being in a lower (i.e., worse) EULAR response category (*P* = 0.003) following adjustment for sex and clinical variables (i.e., baseline DAS28, concurrent DMARD use, and baseline Health Assessment Questionnaire score 29) (further information is available upon request from the corresponding author).

**Table 3:** Methylation quantitative trait locus analyses in 56 patient samples^a^

SNP	CpG locus	*P*	*P*, FDR‐adjusted	Beta^b^
rs3468	cg04857395	2.63 × 10^−7^	0.002	0.64
rs3468	cg26401028	1.05 × 10^−6^	0.006	0.65
rs6850908	cg04857395	8.35 × 10^−6^	0.03	0.66
rs1049574	cg04857395	8.35 × 10^−6^	0.03	0.66

a FDR = false discovery rate.

b Change in methylation M values due to allele dose at the single‐nucleotide polymorphism (SNP) loci tested.

## DISCUSSION

In this epigenome‐wide investigation of DNA methylation as a biomarker of response to TNFi treatments in RA, we have identified 5 differentially methylated sites associated with response to etanercept with test statistics exceeding the 5% FDR threshold. Technical validation using an alternative independent methylation analysis platform (pyrosequencing) indicated that the findings were unlikely to have arisen due to experimental artifact.

The most compelling evidence for differential methylation was observed within exon 7 of the *LRPAP1* gene located on chromosome 4. *LRPAP1* is highly expressed in mononuclear blood cells 30 and encodes a chaperone of low‐density lipoprotein receptor–related protein 1, which is known to influence transforming growth factor β activity 31. In the current study, 4 CpG within exon 7 of *LRPAP1* were observed to be more methylated in nonresponders than in good responders. Exon 7 of *LRPAP1* is in a DNase I hypersensitivity region. Furthermore, cg04857395 overlaps with an H3K36me3 histone mark important in regulation of alternative splicing 32 and with a binding site for CCCTC‐binding factor, a methylation‐sensitive transcription suppressor involved in alternative splicing 33. The level of methylation for the top 2 differentially methylated positions correlated with the rs3468 genotype (position 3,505,698 on chromosome 4), with the A allele correlated with more methylation. Importantly, SNP rs3468 was correlated with EULAR response in a large independent replication experiment (*P* = 0.003), greatly adding to the validity of these findings. SNP rs3468 maps to within a 1,032‐bp noncoding RNA (lnc‐LRPAP1‐1:1) overlapping exon 8 of *LRPAP1* (positions 3,505,324–3,506,184 on chromosome 4) and is predicted to affect binding of the microRNA hsa‐miR‐342‐3p 34. It is therefore plausible that the results observed at *LRPAP1* may have a functional consequence; however, this has not been tested. Future experiments including targeted RNA sequencing in relevant cell types within whole blood are needed.

One strength of the current study is the homogeneity of the patient group selected for the discovery investigation. All of these patients were treated with etanercept and all had either an extremely good response or no response to the drug at 3 months of follow‐up despite having similar baseline clinical characteristics. The importance of selecting a group of patients treated with the same biologic drug was illustrated in a recent GWAS in which there was little overlap between the most associated genetic variants predicting response to etanercept, infliximab, and adalimumab 8. Response to each drug may be controlled by different genetic variants; therefore, it is important to consider that epigenetic mechanisms underlying response may also differ between different types of biologic agents.

A further strength of this study is the large sample size of patients with extreme phenotypes of response to etanercept. By using extremes of response, the power to detect differences in baseline methylation in subsequent responders and nonresponders was enhanced. Using calculations presented in ref. 23, we had 80% power to detect a difference in methylation of 7%, which was in accordance with our own estimates (additional information available upon request from the corresponding author) and the observed difference in methylation for the top differentially methylated positions. However, it may be argued that the study was underpowered to identify the small effect sizes seen for genetic associations with response to therapies 35.

We chose to investigate whole blood in the first instance as this would provide the simplest test in the clinical setting; however, differences in DNA methylation patterns between blood cell types have been reported 36, so it is important to consider the impact of differences in blood composition on our results. Using cell composition estimation based on the distribution of methylation in the results from the BeadChips, no significant differences were observed between good responders and nonresponders, and adjustment for cell composition did not change our results. However, as stated above, cell‐specific experiments may allow finer exploration of the mechanism by which epigenetic changes affect treatment response.

DNA methylation has been implicated in autoimmune disorders in several studies in recent years, but differentiating cause and effect in cross‐sectional studies is difficult. The longitudinal nature of this treatment response study reduces that bias. There is an increasing interest in DNA methylation as a source of biomarkers of response to drug therapies in many diseases, including cancer 37, 38. To our knowledge, ours is the first study to investigate the influence of epigenetic variation on response to biologic therapies. Given the substantial cost and long‐term nature of treatment with these drugs, selecting the right therapy as the first‐choice biologic agent for patients with RA is increasingly important, particularly as biologic therapies targeting other pathways are now available.

In conclusion, it is apparent that treatment response is multifactorial and that a predictive algorithm incorporating assessments of a panel of biomarkers, which could include epigenetic, genetic, and transcriptomic factors and serology, will be necessary to allow prediction of response. Although validation in a larger independent cohort is needed before transfer to clinical practice would be possible, these results indicate that DNA methylation profiling may provide a new biomarker of response to etanercept in patients with RA.

## AUTHOR CONTRIBUTIONS

All authors were involved in drafting the article or revising it critically for important intellectual content, and all authors approved the final version to be published. Dr. Barton had full access to all of the data in the study and takes responsibility for the integrity of the data and the accuracy of the data analysis.

### Study conception and design

Plant, Webster, Smith, Eyre, Hyrich, Morgan, Isaacs, Worthington, Barton.

### Acquisition of data

Plant, Webster, Nair, Oliver.

### Analysis and interpretation of data

Plant, Webster, Hyrich, Wilson, Morgan, Isaacs, Worthington, Barton.
